# Diagnostic Performance of a Novel Ultra-Thin Endoscopy under Narrow-Band Imaging for Superficial Squamous Cell Carcinoma of the Pharynx and Esophagus

**DOI:** 10.3390/cancers16030529

**Published:** 2024-01-26

**Authors:** Akira Dobashi, Yuko Hara, Hiroto Furuhashi, Hiroaki Matsui, Naoya Tada, Mamoru Ito, Toshiki Futakuchi, Masakuni Kobayashi, Shingo Ono, Daisuke Aizawa, Takashi Yamauchi, Machi Suka, Kazuki Sumiyama

**Affiliations:** 1 Department of Endoscopy, The Jikei University School of Medicine, Tokyo 105-8461, Japanfutakuchi.t@gmail.com (T.F.); kaz_sum@jikei.ac.jp (K.S.); 2 Department of Pathology, The Jikei University School of Medicine, Tokyo 105-8461, Japan; 3 Department of Public Health and Environmental Medicine, The Jikei University School of Medicine, Tokyo 105-8461, Japan; yamauchi-t@jikei.ac.jp (T.Y.); suka@jikei.ac.jp (M.S.)

**Keywords:** esophageal squamous cell carcinoma, pharyngeal squamous cell carcinoma, ultra-thin endoscopy, magnifying endoscopy, esophagus, pharynx

## Abstract

**Simple Summary:**

A newly developed ultra-thin endoscope (UTE) provides higher-resolution images, allowing for the evaluation of microvascular patterns. This prospective study aimed to demonstrate the diagnostic performance of UTE for superficial squamous cell carcinoma (SSCC) in the pharynx and esophagus. The Japan Esophageal Society classification is the most common diagnostic system for SESCC. This system requires a precise evaluation of microvessels under magnification with narrow-band imaging. We confirmed that the UTE exhibited sufficient diagnostic performance for the detection and diagnosis of SSCC compared to magnifying endoscopy. The study results showed that most lesions can be accurately diagnosed with high confidence without magnification (i.e., UTE), and the confidence level did not improve, even when the lesion was observed under magnification. UTE, which provides higher-resolution images, can be a reliable tool for the screening or surveillance of SSCC in the pharynx and esophagus.

**Abstract:**

This study aimed to evaluate the diagnostic utility of the ultra-thin endoscope (UTE) for superficial squamous cell carcinoma (SSCC) compared to magnifying endoscopy (ME) under narrow-band imaging. Participants underwent endoscopic examination, and images of pharyngeal and esophageal SCCs, as along with suspicious SSCC lesions, were collected using UTE and ME on the same day. Three image catalogs (UTE, ME-1, and ME-2) were created and reviewed by three expert endoscopists. ME-1 and ME-2 contained the same endoscopic images. The primary endpoint was the intra-observer agreement for diagnosing SCC. Eighty-six lesions (SCC = thirty-nine, non-SCC = forty-seven) in 43 participants were identified. The kappa values for the intra-observer agreement between UTE and ME-1 vs. the control (ME-1 vs. ME-2) were 0.74 vs. 0.84, 0.63 vs. 0.76, and 0.79 vs. 0.88, respectively. The accuracies for diagnosing SCC by UTE and ME-1 were 87.2% vs. 86.0%, 78.0% vs. 73,2%, and 75.6 vs. 82.6%, respectively, with no significant differences (*p* > 0.05). The rates of lesions that were diagnosed with confidence by UTE and ME-1 were 30.2% vs. 27.9%, 55.8% vs. 62.8%, and 58.1% vs. 55.8%, respectively. UTE demonstrates substantial diagnostic performance for SSCC in the pharynx and esophagus.

## 1. Introduction

Of the malignancies arising from the anatomic sites comprising the upper aerodigestive tract, namely the pharyngeal and esophageal carcinomas, histologically, the most common worldwide is squamous cell carcinoma (SCC) [1]. The prognosis of pharyngeal and esophageal carcinomas is poor when detected at advanced stages [2,3,4]. However, when a lesion is detected at an early stage, it can be completely treated with minimally invasive therapeutic procedures, such as endoscopic resection [5,6,7,8,9,10,11,12]. Therefore, early detection of pharyngeal or esophageal carcinomas is essential for a better prognosis, as well as for maintaining an acceptable quality of life.

Narrow-band imaging (NBI) enables the early detection of these carcinomas [13,14]. The detectability of NBI for esophageal squamous SCC (ESCC) is promising, and NBI is now the standard for endoscopic examination [15,16,17]. Magnifying endoscopy (ME) is subsequently performed for differentiation. The Japan Esophageal Society (JES) classification is a useful tool for enabling endoscopists to diagnose lesions as non-SCC (type A) or SCC (type B) in real time by evaluating the change in microvessels on the surface of the lesion using ME with NBI (ME-NBI) [18,19,20]. However, the tip of the ME is wider than that of a routine endoscope, posing a difficulty for nonexpert endoscopists in manipulating the zoom lever [21]. In addition, the classification system requires the evaluation of the presence or absence of four endoscopic findings of microvascular irregularity under magnification: dilatation, tortuosity, irregular changes in the caliber, and various shapes.

To facilitate an accurate diagnosis and avoid overlooking superficial SCC (SSCC), the use of thicker endoscopes with higher resolution and zoom capabilities is needed. Although observations with a conventional ultra-thin endoscope (UTE) can alleviate patient discomfort, the low resolution of conventional UTEs has prevented them from being compared to MEs in the past. Recently, the next-generation UTE with a diameter of 5.8 mm was developed, and the image quality and brightness were considerably improved when compared to those of previous generations of UTEs [22]. A UTE can detect changes in the microvessels owing to the higher resolution of images when combined with NBI. With the introduction of the newly developed high-resolution UTE, there is a potential for obtaining sufficient diagnostic capability even with non-magnified observations. However, studies comparing ME and UTE are lacking, and whether the diagnostic capability of UTE is adequate compared to ME should be determined. Furthermore, if UTE has sufficient diagnostic ability, it can replace detailed examinations. In screening examinations, which are often performed without sedation, not only can patient discomfort be reduced but the number of biopsies required to exclude pharyngeal and esophageal cancers can also be minimized, resulting in significant benefits.

We hypothesized that UTE possesses a sufficient diagnostic performance for SSCC in the pharynx and the esophagus. Thus, we performed endoscopies on participants with pharyngeal or esophageal neoplasms, including SCC, using both UTE and ME in this novel study to compare the diagnostic performance of the UTE with that of ME.

## 2. Materials and Methods

### 2.1. Trial Design and Study Population

A prospective, nonrandomized, single-center trial was conducted at Jikei University Hospital between November 2020 and October 2021. Endoscopies were prospectively performed, and endoscopic images of SCC and suspicious SCC lesions of the pharynx and esophagus were collected. Subsequently, we created an image catalog for the image assessment study. This image catalog included the images obtained from the two different types of endoscopies, UTE and ME. To demonstrate the equivalence of the diagnostic capabilities between UTE and ME in the diagnosis of SSCC, ideally, patients should be assigned into two groups, one undergoing ME and the other undergoing UTE, and then, their diagnostic performance should be compared. However, there is currently no prospective study demonstrating the diagnostic performance of SSCC in the pharynx and esophagus using diagnostic data obtained through UTE. Therefore, as a first step, we decided to conduct an image testing of UTE and ME, which is feasible and lessens the burden on patients, with the aim of demonstrating the diagnostic accuracy of UTE and its ability to provide sufficient diagnostic performance.

### 2.2. Participants

Participants who met the inclusion criteria with a present history of superficial neoplasms, including SCC or an intraepithelial neoplasia (IN) of the pharynx or esophagus or both, were recruited. Participants aged <20 years, those with a history of having received radiotherapy where the lesion was located in the irradiation field, participants in whom stopping antiplatelet or anticoagulant medication was contradicted, those who were currently pregnant, and participants who were evaluated as inappropriate for the research were excluded.

### 2.3. Endoscopy Equipment and Setting

**Figure 1:**
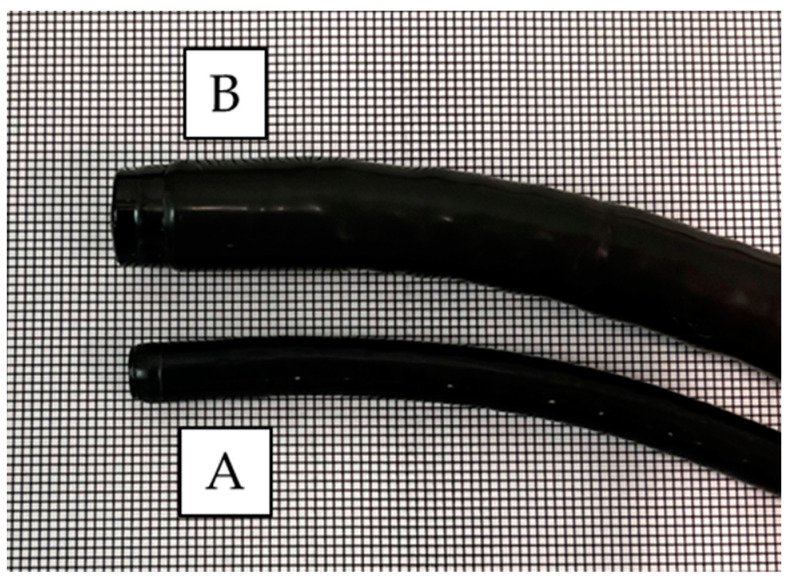
Comparison of the tip between the ultra-thin endoscope and magnifying endoscope. The diameter of the ultra-thin endoscope is 5.8 mm (A), whereas that of the magnifying endoscope is 9.9 mm (B).

A video endoscopy system (EVIS X1; Olympus, Tokyo, Japan) was used for both UTE (GIF-1200N) and ME (GIF-EZ1500 or GIF-XZ1200; Olympus) (Figure 1). Regarding the image sensor, both endoscopes use a complementary metal oxide semiconductor, which is expected to improve the image quality compared to a charge-coupled device. The diameters of the UTE and ME were 5.8 and 9.9 mm, respectively. A black rubber cap (MAJ-1989; Olympus) was mounted on the ME tip. The structural enhancement function for image enhancement was set to the A8 mode in the NBI, as per our previous study [23].

### 2.4. Endoscopic Procedure

**Figure 2:**
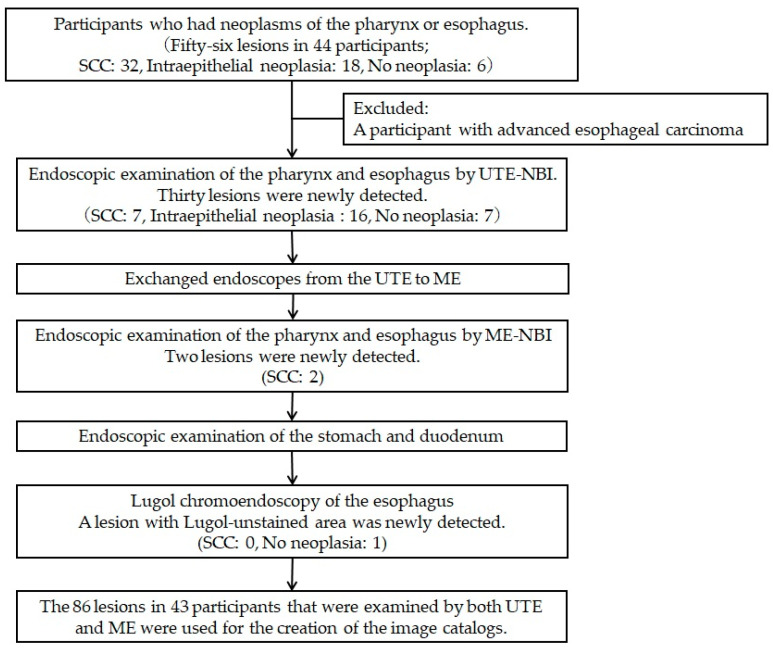
Flowchart of the study procedures. We recruited 44 participants, and 1 participant was excluded because a lesion that had been previously diagnosed as superficial esophageal cancer was diagnosed at an advanced stage during the endoscopy. Eighty-six lesions in 43 participants were examined by both ultra-thin endoscopy with narrow-band imaging (UTE-NBI) and magnifying endoscopy with narrow-band imaging (ME-NBI), and these lesions were used for the image assessment study. A histopathological analysis revealed squamous cell carcinoma (SCC) in 39 lesions, intraepithelial neoplasia in 34 lesions, and no neoplasia in 13 lesions.

**Figure 3:**
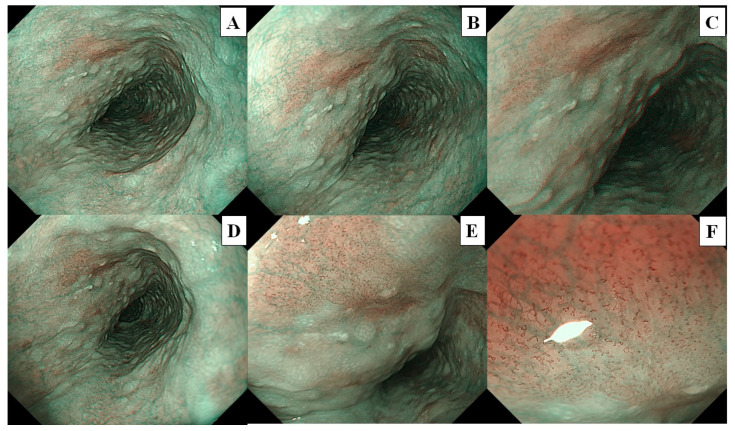
The lesion is a typical case of non-squamous cell carcinoma, and histopathological analysis revealed intraepithelial neoplasia. These images were used for the image assessment study. (**A**) Far view, (**B**) near view, and (**C**) near view plus 1.6× electrical magnification were photographed by ultra-thin endoscopy. (**D**) Far view, (**E**) half-zoom view (about 60× optical magnification), and (**F**) full-zoom view (125× optical magnification) were photographed by magnifying endoscopy.

**Figure 4:**
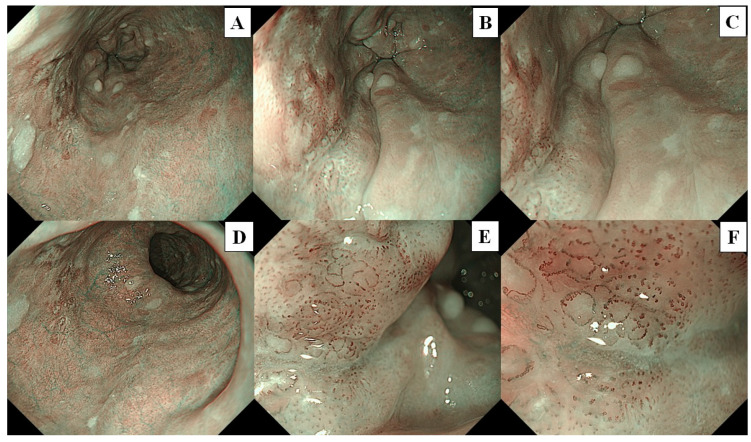
The lesion is a typical case of squamous cell carcinoma. These images were used for the image assessment study. (**A**) Far view, (**B**) near view, and (**C**) near view plus 1.6× electrical magnification were photographed by ultra-thin endoscopy, (**D**) Far view, (**E**) half-zoom view (about 60× optical magnification), and (**F**) full-zoom view (125× optical magnification) were photographed by magnifying endoscopy.

A flowchart of the study procedure is presented in Figure 2. First, an endoscopist confirmed the presence of neoplasms that had been identified in the pharynx or esophagus, as per previous endoscopic reports. Endoscopic examination of the pharynx and esophagus by UTE with NBI (UTE-NBI) was performed. Once the endoscopist detected target lesions that had been preidentified upon previous endoscopies or newly suspicious SCC lesions during the current trial, these lesions were carefully photographed in NBI mode. Three images were recorded that included far (Figure 3A and Figure 4A), near (Figure 3B and Figure 4B), and near plus electronic magnification (1.6×) (Figure 3C and Figure 4C) views. The endoscopes were then exchanged from the UTE to ME.

Similarly, the endoscopist examined the pharynx and esophagus with ME-NBI and endoscopic images that included far (without magnification; Figure 3D and Figure 4D), half-zoom (about 60× optical magnification; Figure 3E and Figure 4E), and full-zoom views (125× optical magnification; Figure 3F and Figure 4F). Finally, Lugol chromoendoscopy was performed in the esophagus, and the endoscopist biopsied a newly detected lesion, which had an irregular shape and was ≥5 mm in size, with a Lugol-unstained area. All procedures were performed by a single expert endoscopist (A.D.) who had experience diagnosing more than a thousand cases of superficial pharyngeal SCC and ESCC and was certified by the Japan Gastroenterological Endoscopy Society Board.

Suspicious SCC lesions were defined as macroscopic morphological changes on the mucosal surface or as having the appearance of a well-demarcated brownish area ≥5 mm in diameter on non-magnified NBI. The histopathology of the biopsied specimens or resected lesions was used as a reference.

### 2.5. Image Assessment Study

#### Preparation of Image Catalogs

Three sets of image catalogs were created. The Test-1 catalog contained endoscopic images photographed using the UTE-NBI. The Test-2 and Test-3 catalogs contained the same endoscopic images photographed by the ME-NBI. Each lesion had three images that included far, near, and near plus electronic magnification in UTE (Figure 3A–C and Figure 4A–C) and far, half-zoom, and full-zoom (Figure 3D–F and Figure 4D–F) in ME. The endoscopic images were arranged according to random number tables created in Excel (Office 365; Microsoft, Seattle, WA, USA). Three images of the same lesion were arranged consecutively.

### 2.6. Assessment of the Images

**Figure 5:**
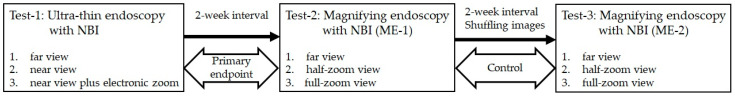
Process of the image assessment study. The three reviewers evaluated the three sets of image catalogs and determined the diagnosis of squamous cell carcinoma or non-squamous cell carcinoma with confidence levels (high or low). Test-1 contains images obtained with ultra-thin endoscopy with narrow-band imaging (UTE-NBI). Test-2 and Test-3 contain images obtained with magnifying endoscopy with narrow-band imaging (ME-NBI). Test-2 and Test-3 include the same images, but the order differs. All the catalogs contain 258 images of 86 lesions.

Three expert endoscopists with experience in diagnosing superficial pharyngeal SCC and ESCC in more than 300 cases reviewed the images. The reviewer evaluated whether the lesions were SCC or non-SCC. The confidence level of the diagnosis was also evaluated. High confidence was defined as the ability of the reviewer to observe or treat the lesion based only on endoscopic imaging without a biopsy. They assessed the three catalogs at 2-week intervals to cleanse their memories (Figure 5). The reviewers evaluated the lesions according to the simplified dyad criteria [23,24] for UTE-NBI and JES criteria [18] for ME-NBI. When the microvessels on the lesion showed proliferation or irregular changes (corresponding to type B1 in the JES criteria), changes without a loop-like formation (corresponding to type B2 in the JES criteria), or changes with highly dilated vessels (corresponding to type B3 in the JES criteria) under UTE-NBI examination, the lesion was diagnosed as SCC in Test-1. When the microvessels of the lesion revealed type B based on the JES criteria (type B1, all the endoscopic changes of microvascular irregularity with dilatation, tortuosity, irregular changes in the caliber, and various shapes; type B2, type B vessels without a loop-like formation; type B3, highly dilated vessels in which the calibers appear to be more than three times that of the usual B2 vessels), the reviewer diagnosed the lesion as SCC in Test-2 (ME-1) and Test-3 (ME-2), which contained images photographed using ME-NBI.

### 2.7. Outcomes

The primary endpoint was the intra-observer agreement and rate of concordance for diagnosing SCC between UTE (Test-1) and ME-1 (Test-2). The results of the intra-observer agreement between ME-1 (Test-2) and ME-2 (Test-3) were used for the control. The secondary endpoints included the diagnostic performance of each endoscope for diagnosing SCC, the rate of lesions that were diagnosed with high confidence levels, and the undetectable SCC rate by UTE during the endoscopic procedure.

### 2.8. Sample Size

According to previous studies, the sensitivity of ME-NBI for the diagnosis of pharyngeal SCC and ESCC is 90% [13,21,23]. We estimated that the sensitivity of UTE-NBI for the diagnosis of pharyngeal SCC and ESCC would be 80%. The sample size of the lesions for the primary endpoint was calculated to be 80 if the true Cohen’s kappa statistic was 0.9; the kappa value under the null hypothesis was 0.7, and the probability for an alpha error and power (reflecting a beta error of 0.2) would be 0.05 and 0.80, respectively. Our prospective study showed that the average number of SCC or suspicious SCC lesions was approximately two in a participant who possessed at least one SCC or suspicious SCC lesion before endoscopy. Thus, the total sample size was calculated to be 44 participants, with a predicted 10% of the participants not attending every planned endoscopic examination. 

### 2.9. Pathological Diagnosis

Histopathological analysis and diagnosis were performed by an expert gastrointestinal pathologist (D.A.) who was blinded to the endoscopic images. Each specimen was graded as non-neoplastic (including normal mucosa, esophagitis, and atypical epithelium); IN; or SCC. SSCC was defined as invasion up to the subepithelial layer of the pharynx and the submucosal layer of the esophagus. If the histopathological results of a lesion differed between the biopsied and resected specimens, the more malignant histopathological result was considered.

### 2.10. Statistical Analysis

The intra-observer agreement for each reviewer was shown based on kappa values. The kappa values were interpreted according to the evaluation proposed by Landis and Koch: poor (kappa < 0.00), slight (0.00 ≤ kappa ≤ 0.20), fair (0.21 ≤ kappa ≤ 0.40), moderate (0.41 ≤ kappa ≤ 0.60), substantial (0.61 ≤ kappa ≤ 0.80), and almost perfect (0.81 ≤ kappa ≤ 1.00). The sensitivity, specificity, positive predictive value (PPV), negative predictive value (NPV), and overall accuracy for diagnosing SSCC of the pharynx and esophagus in the UTE and ME-1 were calculated for the diagnostic yields. McNemar’s test was used to compare the diagnostic accuracy of UTE and ME-1 for all lesions among the reviewers. Furthermore, the rate of lesions, sensitivity, specificity, PPV, NPV, and overall accuracy were compared between the UTE and ME-1 for lesions with high confidence levels using the chi-squared test or Fisher’s exact test. The undetectable SCC rate was calculated as the number of lesions that were overlooked by the UTE compared to the number of lesions that were detected by the ME-NBI or Lugol chromoendoscopy during the endoscopic examination.

The statistical significance was set at *p* < 0.05. Statistical analyses were performed using Stata version 14.2 (Stata Corp., College Station, TX, USA).

## 3. Results

Forty-four participants who met the inclusion criteria underwent endoscopy in this study. One participant was excluded after the endoscopy, because a lesion that had been previously diagnosed as superficial esophageal cancer was diagnosed at an advanced stage during the endoscopy. Therefore, we used the images obtained from 43 participants.

We analyzed the images obtained from 43 participants. The participant age ranged from 45 to 87 years, with a median age of 70 years, and the male-to-female ratio was 36:7. Neoplasms were identified in the pharynx or esophagus, as per previous endoscopic reports. The predefined neoplasms for endoscopy included pharyngeal lesions in 13 participants, esophageal lesions in 27 participants, and both pharyngeal and esophageal lesions in 3 participants.

We used dual-focus ME (GIF-EZ1500) for the first 3 participants and zoom-type ME (GIF-XZ1200) for the remaining 40 participants. We examined 56 preidentified suspicious SCC lesions and photographed them using both UTE-NBI and ME-NBI (Figure 1). During this study, 30 lesions were newly detected by UTE-NBI, and upon histopathological analysis, 7 lesions were diagnosed as SCCs and 23 lesions were diagnosed as non-SCCs (IN, 16; no neoplasia, 7) (Figure 2). Following the examination, two SCCs were newly detected upon examination by ME-NBI. However, those two cases had some synchronous SCCs that were detected by UTE-NBI in the pharynx and esophagus, and those overlooked SCCs were less malignant than the others that were detected by UTE-NBI. No SCC was detected using Lugol chromoendoscopy alone. The undetectable SCC rate for UTE-NBI was calculated as 4.4% (2/45). 

Finally, we created image catalogs, incorporating 86 lesions. The histopathological analysis revealed that 39 lesions were diagnosed as SCCs, and 47 lesions were diagnosed as non-SCCs. The characteristics of the lesions are presented in Table 1. The median lesion size was 8 mm in diameter, and 52.3% of the lesions were a macroscopically completely flat (0-IIb) type.

### 3.1. Primary Outcome

The results of the primary endpoints are presented in Table 2. The kappa values (rate of the same diagnosis) in the three reviewers for intra-observer agreement between UTE (Test-1) and ME-1 (Test-2) vs. the control (Test-2 vs. Test-3) were 0.74 (87.2%) vs. 0.84 (91.9%), 0.63 (81.4%) vs. 0.76 (88.3%), and 0.79 (90.7%) vs. 0.88 (94.2%), respectively. The kappa values in UTE vs. ME-1 were interpreted as substantial by all reviewers, and those in ME-1 vs. ME-2 were interpreted as substantial to almost perfect.

### 3.2. Diagnostic Performance of UTE-NBI and ME-NBI

The diagnostic performance of each endoscope is presented in Table 3. Each value was approximately equal to that of the other endoscopes, with no statistically significant differences (*p* > 0.05). The rates of the lesions that were diagnosed with confidence by UTE and ME-1 were 30.2% vs. 27.9%, 55.8% vs. 62.8%, and 58.1% vs. 55.8%, respectively. The accuracies for diagnosing SCC in the high confidence lesions by UTE and ME-1 were 96.2% vs. 95.8%, 93.8% vs. 85.2%, and 86.0 vs. 87.5%, respectively, with no significant differences (*p* > 0.05).

In the subgroup analysis based on the lesion location, there was no difference in the diagnostic performance between UTE and ME, except for the diagnosis of the esophagus by reviewer A (Table 4). Reviewer A had only one esophageal lesion that was evaluated as SCC by UTE and non-SCC by ME, but there were no fewer than nine esophageal lesions that were evaluated as non-SCC by UTE and SCC by ME. From those results, the sensitivity increased and specificity decreased in the diagnosis of ME compared to UTE in the esophageal lesions by reviewer A.

## 4. Discussion

The present study revealed that the diagnostic performance of UTE-NBI for SSCC of the pharynx and esophagus was comparable to ME-NBI, even though UTE does not have a magnifying function and its diameter is thinner. The image quality of UTE-NBI may be adequate to achieve a precise diagnosis of SSCC. The detection rate of SSCC of the pharynx and esophagus by UTE-NBI was considerable at 95.6% (43/45). 

Inoue et al. first reported that changes in the microvessels of superficial esophageal lesions could be used for the differential diagnosis of esophageal lesions [25]. Subsequently, the JES classification system was developed [18] and is now widely used in daily practice. We previously reported on the diagnostic practicality of the simplified dyad criteria for the detection of SCC, with the intent of conforming the acceptability and ease of the criteria to a nonexpert setting [23]. In a previous study, to demonstrate the effectiveness of the simplified dyad criteria, we used dual-focus endoscopy, with an optical magnification of approximately 40 times, lower than that of a conventional ME (90× optical magnification; GIF-H290Z; Olympus). Furthermore, we revealed that the combination of the simplified dyad criteria and examination by dual-focus endoscopy has sufficient diagnostic performance for the diagnosis of SCC. From the results, we considered that a higher magnification might not be needed for the differential diagnosis [23]. Thus, we believe that the combination of the simplified dyad criteria and high-resolution UTE could be an option for a reliable endoscopic examination of the pharynx and esophagus.

The accuracy of predicting the histopathology with NBI-based diagnostic systems, such as the JES classification and the simplified dyad criteria, is not perfect, and a precise diagnosis may be difficult for some lesions. The disadvantage of NBI-based diagnostic systems is that we cannot directly observe the changes in the nuclei or histological structures, which pathologists use to make a diagnosis. SSCC is accompanied by structural changes at the histological level, and SCC cells cause morphological changes in intra-papillary capillary loops (IPCL), which are located on the surface of the squamous layer [26]. The NBI-based diagnostic systems were evaluated based on the degree of microvascular changes. A discrepancy exists between the changes in histology and those in the IPCL. Therefore, the accuracy of the SCC diagnosis is limited. In contrast, endocytoscopy [27,28] and probe-based confocal laser endomicroscopy (p-CLE) [29] can directly observe the histology in vivo, and the diagnostic system is basically the same as that for the pathological analysis. The diagnostic performance of endocytoscopy and p-CLE has been hypothesized to be better than that of NBI, but these modalities require preparation by spraying or injecting a dye. The advantage of the diagnostic system with NBI is that it is quick and easy, and the modality can be changed from white light imaging by simply pushing the button.

ME has been used to detect slit changes in IPCL and diagnose SCC in vivo [18]. However, high-power magnification may improve or worsen the process of obtaining a diagnosis. As previously mentioned, certain lesions pose a challenge for NBI-based diagnostic systems, as they cannot diagnose non-SCC due to significant changes in the IPCL under high-power magnification and vice versa, as shown by the result of reviewer A in the subgroup analysis. The ratio of high confidence levels between UTE and ME in this study was approximately the same. This means that endoscopists can evaluate changes in microvessels without high-power magnification and that the add-on effect of high-power magnification may be limited. The high-resolution images obtained by UTE may reach the threshold for the precise diagnosis of SCC, and it may not be reasonable to magnify with an excessive power for microvessels [21]. The diagnostic system with the simplified dyad criteria [23] and examination by UTE may be well balanced, with a satisfactory diagnostic performance and a simple algorithm, in addressing the disadvantages of the JES classification with ME. 

In the field of otorhinolaryngology, a rigid or a flexible nasopharyngeal videoscope is used for the diagnosis of pharyngeal cancer, and the outer diameter is approximately 4 mm and is thinner than that used in esophagogastroduodenoscopy [30]. With the same situation as esophagogastroduodenoscopy, NBI led to an improvement in the early detection of pharyngeal cancer [31,32], and the detection rate of a nasopharyngeal videoscope with NBI and high-definition television was reported as significantly higher than that of white light imaging. The sensitivity and specificity of upper aerodigestive tract cancer, including oral cavity, oropharynx, larynx, and hypopharynx, by NBI with high-definition television were reported as 98% and 96%, respectively [33]. This study was a patient-based analysis, and the biopsy protocol and lesion size were not described. A previous study that provided the diagnosis was performed using the criteria based on changes in the IPCL with a nasopharyngeal videoscope and NBI [34]. However, there were no standardized diagnostic criteria for diagnosing pharyngeal SCC by a nasopharyngeal videoscope with NBI. No magnifying nasopharyngeal videoscope is available at the moment, but a report showed that contact endoscopy improved the diagnostic performance [35]. However, contact endoscopy needs a rigid endoscope. Generally, the superficial layer of malignant lesions is fragile and quickly bleeds as soon as it makes contact. Once bleeding commences, observation under NBI with magnification becomes difficult, and the indication of contact endoscopy should be limited.

On the issue of the field cancerization phenomenon, a patient who has a history of ESCC needs screening of the pharynx and vice versa [36]. A study reported that pharyngeal SCC was detected in 8.4% of patients with ESCC [37]. Meanwhile, ESCC was detected in 14.3% of patients with pharyngeal cancer. The advantage of esophagogastroduodenoscopy is that patients can be inspected from the oral cavity to the esophagus. Thus, before the treatment of pharyngeal or esophageal cancer, endoscopists should screen and observe the pharynx and esophagus. 

This study had some limitations. The endoscopic examination was conducted by a single expert endoscopist, and the ease of endoscopic maneuvers by nonexperts is unknown. However, UTE is believed to be more advantageous, because a smaller diameter is acceptable to both patients and endoscopists [38]. A hood at the tip of the endoscope has been used only for ME, because a hood for UTE is not currently available. The hood was helpful in observing the lesions in a narrow area, such as the pyriform sinus and the esophagogastric junction. The hood may affect the undetectable SCC rate or endoscopic image quality. Endoscopic imaging is also affected by peristalsis, particularly for esophageal lesions. A prospective trial with a real-time diagnostic evaluation is required to address this issue. The reviewing endoscopists could not be blinded in the evaluation of two types of endoscopes (UTE or ME). This bias cannot be completely eliminated, even in a prospective randomized trial.

## 5. Conclusions

UTE has considerable benefits, including a small diameter acceptable to both patients and endoscopist, not requiring magnification, and demonstrating a high diagnostic performance for SSCC of the pharynx and esophagus comparable to ME. Owing to its considerable detection rate, UTE can be a reliable tool for the screening or surveillance of high-risk patients with pharyngeal and esophageal SCC.

## Figures and Tables

**Table 1:** Characteristics of the lesions for the image assessment study (n = 86).

Location: oropharynx, hypopharynx/Ce, Ut, Mt, Lt, Ae	3, 19/12, 19, 23, 10, 0
Diameter (mm), median (range)	8 (3–54)
Macroscopic type: 0-I/0-IIa, 0-Iib, 0-Iic/0-III	1/10, 45, 30/0
Histopathology: SCC, IN, no neoplasia	39, 34, 13

Ce, cervical esophagus; Ut, upper thoracic esophagus; Mt, middle thoracic esophagus; Lt, lower thoracic esophagus; Ae, abdominal esophagus; SCC, squamous cell carcinoma; IN, intraepithelial neoplasia.

**Table 2:** Kappa values (rate of the same diagnosis) of diagnosing squamous cell carcinoma between UTE and ME by the three reviewers (intra-observer agreement).

	UTE (Test-1) vs. ME-1 (Test-2)	ME-1 (Test-2) vs. ME-2 (Test-3)
Reviewer A	0.74 (87.2%)	0.84 (91.9%)
Reviewer B	0.63 (81.4%)	0.76 (88.3%)
Reviewer C	0.79 (90.7%)	0.88 (94.2%)

Test-2 and Test-3 contained the same endoscopic images. UTE, ultra-thin endoscopy; ME, magnifying endoscopy.

**Table 3:** Diagnostic performance of each endoscope for all the lesions and high confidence lesions (UTE vs. ME).

	All Lesions (n = 86)				High Confidence Lesions			
		UTE	ME-1	*p*-Value		UTE	ME-1	*p*-Value
Reviewer A					Number of lesions	26 (30.2%)	24 (27.9%)	0.74 **
	Sensitivity (%)	74.4	84.6		Sensitivity (%)	95.7	100	0.32 **
	Specificity (%)	97.9	87.2		Specificity (%)	100	0	0.046
	Accuracy (%)	87.2	86.0	0.059 *	Accuracy (%)	96.2	95.8	0.95 **
	PPV (%)	96.7	84.6		PPV (%)	100	95.8	0.33 **
	NPV (%)	82.1	87.2		NPV (%)	75.0	−	−
Reviewer B					Number of lesions	48 (55.8%)	54 (62.8%)	0.35 **
	Sensitivity (%)	79.5	82.1		Sensitivity (%)	95.2	92.9	0.73 **
	Specificity (%)	76.0	66.0		Specificity (%)	92.6	76.9	0.11 **
	Accuracy (%)	78.0	73.2	0.13 *	Accuracy (%)	93.8	85.2	0.16 **
	PPV (%)	73.8	66.7		PPV (%)	90.9	81.3	0.33 **
	NPV (%)	81.8	81.6		NPV (%)	96.2	90.9	0.45 **
Reviewer C					Number of lesions	50 (58.1%)	48 (55.8%)	0.76 **
	Sensitivity (%)	59.0	69.2		Sensitivity (%)	87.5	81.5	0.33 **
	Specificity (%)	89.4	93.6		Specificity (%)	84.6	95.2	0.36 **
	Accuracy (%)	75.6	82.6	0.48 *	Accuracy (%)	86.0	87.5	0.83 **
	PPV (%)	82.1	90.0		PPV (%)	88.0	95.7	0.34 **
	NPV (%)	72.4	78.6		NPV (%)	84.0	80.0	0.71 **

* McNemar’s test; ** chi-squared test or Fisher’s exact test. UTE, ultra-thin endoscopy; ME, magnifying endoscopy; PPV, positive predictive value; NPV, negative predictive value; NS, not significant.

**Table 4:** Diagnostic performance of each endoscope for all the lesions based on the lesion location (UTE vs. ME).

	Pharynx (n = 22)				Esophagus (n = 64)			
		UTE	ME-1	*p*-Value		UTE	ME-1	*p*-Value
Reviewer A	Sensitivity (%)	92.3	100	1.00 *	Sensitivity (%)	65.4	76.9	0.011 *
	Specificity (%)	100	100		Specificity (%)	97.4	84.2	
	Accuracy (%)	95.5	100		Accuracy (%)	84.3	81.3	
	PPV (%)	100	100		PPV (%)	94.4	76.9	
	NPV (%)	90.0	100		NPV (%)	80.4	84.2	
Reviewer B	Sensitivity (%)	84.6	92.3	0.317 *	Sensitivity (%)	76.9	76.9	0.248 *
	Specificity (%)	55.6	44.4		Specificity (%)	81.6	71.1	
	Accuracy (%)	72.7	72.7		Accuracy (%)	79.7	73.4	
	PPV (%)	73.3	70.6		PPV (%)	74.1	64.5	
	NPV (%)	71.4	80.0		NPV (%)	83.8	81.8	
Reviewer C	Sensitivity (%)	84.6	84.6	0.157 *	Sensitivity (%)	46.2	61.5	0.103 *
	Specificity (%)	66.7	88.9		Specificity (%)	94.7	94.7	
	Accuracy (%)	77.2	86.4		Accuracy (%)	75.0	81.2	
	PPV (%)	78.6	91.7		PPV (%)	85.7	88.9	
	NPV (%)	75.0	80.0		NPV (%)	72.0	78.2	

* McNemar’s test. UTE, ultra-thin endoscopy; ME, magnifying endoscopy; PPV, positive predictive value; NPV, negative predictive value.

## Data Availability

The data are available upon request to the co-author (A.D.).

## Abbreviations

SCC, squamous cell carcinoma; NBI, narrow-band imaging; ESCC, esophageal squamous SCC; JES, Japan Esophageal Society; ME-NBI, magnifying endoscopy with NBI; UTE, ultra-thin endoscope; IN, intraepithelial neoplasia; SSCC, superficial squamous cell carcinoma; PPV, positive predictive value; NPV, negative predictive value; IPCL, intra-papillary capillary loops; p-CLE, robe-based confocal laser endomicroscopy.
